# Determination of Elastic Modulus, Stress Relaxation Time and Thermal Softening Index in ZWT Constitutive Model for Reinforced Al/PTFE

**DOI:** 10.3390/polym15030702

**Published:** 2023-01-30

**Authors:** Chuang Chen, Zihan Guo, Enling Tang

**Affiliations:** Key Laboratory of Transient Physical Mechanics and Energy Conversion Materials of Liaoning Province, Shenyang Ligong University, Shenyang 110159, China

**Keywords:** reinforced Al/PTFE active materials, ZWT constitutive model parameters, compressive mechanical properties, temperature, strain rate

## Abstract

Al/PTFE has the advantages of high impact-responsive energy release, appropriate sensitivity, a fast energy release rate, and high energy density, and it is increasingly widely being used in the field of ammunition. In this paper, based on the traditional formula Al/PTFE (26.5%/73.5%), the reinforced Al/PTFE active materials are prepared by the process of cold pressing, sintering, and rapid cooling. Quasi static and dynamic compression experiments were carried out under different compression pressures (200~800 MPa), strain rates (0.002 s^−1^, 0.02 s^−1^, 1400~3300 s^−1^), and temperatures (23 °C, −20 °C, −30 °C, −40 °C). The effects of pressure, strain rate, and temperature on the quasi-static and dynamic compression properties of Al/PTFE materials are analyzed. The results show that the reinforced Al/PTFE specimens show a significant correlation between temperature and strain rate. Based on the classical Zhu–Wang–Tang (ZWT) constitutive model, the ZWT constitutive model parameters of the reinforced Al/PTFE active materials under different pressing pressures at room temperature and the ZWT constitutive model parameters of the reinforced Al/PTFE active materials at low temperature are obtained by fitting, respectively. The accuracy of the constitutive model parameters (elastic modulus, stress relaxation time, and thermal softening index) is verified. In this paper, a constitutive model considering both temperature and strain rate effects is established in order to provide reference for the study of mechanical properties of active materials.

## 1. Introduction

Al/PTFE (aluminum/polytetrafluoroethylene) active materials, as energetic materials with a strong deflagration reaction and strong energy release under impact load, are widely used in ammunitions such as shaped charge liners and warhead shells due to their advantages such as appropriate sensitivity, high energy density and large amount of gas generated by reaction [1]. The strength of traditional Al/PTFE active materials under dynamic loading is only tens of MPa, which restricts the promotion of materials under the demand of high strain rate. In order to greatly improve the dynamic compressive strength of Al/PTFE active materials, the reinforced Al/PTFE are prepared using a combination of cold pressing, sintering, and rapid cooling. Traditional Al/PTFE shows obvious strain hardening and strain-rate strengthening effects in compression experiments, with strain rates ranging from 10^−3^ to 10^4^ [2,3,4]. It is necessary to carry out research on the mechanical properties and constitutive equations of reinforced Al/PTFE prepared by improved process.

Many scholars have constructed Johnson–Cook constitutive equations for Al/PTFE reactive materials based on quasi-static and dynamic compression test data. Xu [5] obtained the compression constitutive equation of Al/PTFE by combining a quasi-static experiment, a dynamic experiment, and microscopic analysis. It was measured that the tensile strength of the traditional formula Al/PTFE (mass ratio 26.5/73.5) active material was 24.9 MPa, and the failure mode of the material under impact loading was analyzed. Shen [6] studied the mechanical response characteristics of Ti/PTFE materials under a wide range of strain rates by SHPB test, and the parameters of the Johnson–Cook constitutive model were obtained by fitting the experimental curves. Liu [7] used copper powder to treat PTFE as a filler in order to obtain modified polytetrafluoroethylene (PTFE/Cu). Quasi static compression experiments and SHPB dynamic compression experiments were carried out on the material to obtain the basic mechanical property parameters and build an elastoplastic constitutive equation. Ding et al. [8] constructed a Johnson–Cook constitutive model of PTFE/Al/Si reactive material considering the strain hardening effect, strain rate hardening effect, and thermal softening effect based on the experimental data of quasi-static and dynamic mechanical properties parameters. Sun et al. [9] determined the parameters of the Johnson–Cook constitutive (A, B, n, C, and m) and failure models (D1~D5) based on the experimental results and numerical iteration; the stress–strain curves are in good agreement with the experimental results. Huang et al. [10] established the Johnson–Cook constitutive model of PTFE/Al/MoO_3_, and the model results agreed well with the experimental data. Zhang et al. [11] proposed a modified Johnson–Cook model considering strain and strain-rate coupling, and the parameters of the modified Johnson–Cook model of Zr/PTFE and Ti/PTFE reactive materials were determined, which can describe and predict plastic flow stress. Zhang et al. [12] determined the Johnson–Cook constitutive constants of PTFE/Al/W, and the predicted stress–strain response at elevated strain rates and temperatures agreed well with the tested results. The determined constitutive models provided good understanding of the constitutive behavior of the materials and corresponding numerical studies. Wu et al. [13] established the Johnson–Cook constitutive model of Al/PTFE, which can characterize well the mechanical response of Al/PTFE over a range of strain rates and temperatures and can supply certain references for the practical applications of this reactive material. Feng et al. [14] based on strain rate sensitivity, a Johnson–Cook constitutive model applicable to Al/PTFE was fitted. Li [15] studied the dynamic mechanical properties of tungsten zirconium active materials using an SHPB experimental device and obtained the dynamic stress–strain curves of tungsten zirconium materials under different strain rates. Based on the one-dimensional elastic brittle damage model, the constitutive equation of tungsten zirconium active materials was obtained by fitting the experimental data. Chen et al. [16] studied the dynamic compressive mechanical properties of tungsten-containing active materials. Based on the modified Johnson–Cook constitutive model, the material parameters were fitted and substituted into the finite element calculation software. The calculation results were in good agreement with the experimental results. Raftenberg et al. [17] studied the dynamic compressive mechanical response of Al/PTFE, and simulated Taylor impact tests at different speeds based on the constitutive equations of Johnson–Cook and the PSDam strength model. Casem [18] obtained the compressive stress–strain curve of Al/PTFE composite in the strain rate range of 10^−3^ to 8000 s^−1^ using the universal testing machine and SHPB system. The dynamic deformation behavior of Al/PTFE was simulated based on Johnson–Book, modified Johnson–Cook constitutive equation and Zerilli Armstrong equation. Zhang [19] prepared Al/PTFE/W specimens with different particle sizes of aluminum powder and studied the quasi-static compression and dynamic mechanical properties of the materials by using the universal material testing machine and the split Hopkinson pressure bar (SHPB) experimental technology. Based on the experimental data, the Johnson–Cook elasto-plastic constitutive model parameters of Al/PTFE/W active materials were obtained by fitting, and the Hopkinson compression bar experiment was simulated by Abquas to verify the correctness of its constitutive equation. However, the Johnson–Cook constitutive model is actually a constitutive model can be summarized as a constitutive model of multiplying strain effect, strain rate effect and temperature effect. The range of application of this model is limited. With the increase of strain rate, the linear relation of flow stress–strain rate logarithm is not satisfied.

In terms of the mechanical properties of polymer materials at high strain rates, Wang et al. [20] established a ZWT nonlinear viscoelastic constitutive model. Studies have shown that this constitutive model is applicable not only to the polymer itself, but also to the polymer matrix composites [21,22,23,24]. Lai et al. [25] introduced the damage function into the ZWT model to describe the constitutive relationship between ultra-high-performance cement-based composites. Wang et al. [26] modified the ZWT constitutive model by introducing strain rate and temperature effects in order to simulate the dynamic behavior of a polymethyl methacrylate (PMMA) aircraft windshield under bird impact. Xu et al. [27] studied the mechanical behavior of liquid nitrile rubber-modified epoxy resin and chose the standard ZWT nonlinear viscoelastic model to predict the elastic behavior of LNBR/epoxy composites under wide ranges of strain. Meanwhile, the authors simulated the mechanical behavior of LNBR/epoxy composites with the model parameters obtained from the experiments. Luo et al. [28] used quasi-static and dynamic compression stress–strain curves to fit the parameters of the Zhu–Wang–Tang (ZWT) constitutive equation at different temperatures. A subroutine for the ZWT constitutive model was developed in ABAQUS, and numerical simulations of split Hopkinson pressure bar tests were performed. Luo et al. [29] studied the dynamic mechanical properties and constitutive model of shale with different bedding under triaxial impact testing and established an improved dynamic constitutive relation of shale based on the ZWT model which combined with the damage theory and simplified the low-frequency term. Dong et al. [30] constructed a dynamic constitutive model of tensile and compressive damage on the basis of the ZWT and statistical damage models; the results show that the constructed dynamic constitutive model of tensile and compressive damage could considerably simulate the tensile and compressive stress–strain relations and failure features of sandstones well. Dar et al. [31] established a ZWT nonlinear viscoelastic constitutive model coupled with temperature and strain rate effects and realized the embedding of the ZWT constitutive model by establishing a user-defined material subprogram in the finite element solver LS-DYNA.

The constitutive model of the Al/PTFE active materials under the influence of low temperature and pressing pressure is still not perfect. In this paper, the dynamic compression strength of Al/PTFE active materials is greatly improved by using the preparation process of cold pressing, sintering, and rapid cooling. Static compression experiments and dynamic compression experiments at different temperatures and different strain rates are carried out on reinforced Al/PTFE specimens prepared under different pressing pressures. The static/dynamic compression mechanical behavior of the material is analyzed, and a ZWT constitutive model considering temperature is established by adding a temperature-related term to the classical ZWT constitutive model and by assuming that the parameters of the constitutive model are temperature dependent in order to describe the mechanical behavior of reinforced Al/PTFE materials at different temperatures and different strain rates. Based on the experimental data, modified ZWT constitutive model parameters suitable for reinforced Al/PTFE active materials are obtained by fitting.

## 2. Experiment

### 2.1. Preparation of Reinforced Al/PTFE

In this paper, based on the traditional formula Al/PTFE (26.5%/73.5%), a reinforced Al/PTFE active material is prepared through improvements to the sintering process and using the rapid cooling method. The ratio and preparation technology of traditional formula Al/PTFE is proposed by Vasant [32].

Al powder is an Al particle produced by Beijing Xinyuan Technology Co., Ltd., and PTFE powder is a PTFE 7A particle produced by DuPont. An electronic balance was used to weigh the Al powder and PTFE according to the required ratio. Due to its small particle size, PTFE easily absorbs moisture and forms white clumps during storage. In order to remove the moisture in the raw material, a vacuum drying oven was used to treat PTFE. The temperature of the vacuum drying oven was set to 60 °C, the vacuum degree was 0.08 MPa, and the duration was 10 h. PTFE and Al were mixed evenly according to the proportion of 26.5%:73.5% of the mass ratio of Al: PTFE, and the mixing time was 10 min. The mixed powder was added into the self-made pressing mould. In order to avoid stratification, the mixed powder was spread in the pressing mould as far as possible by shaking the mould during the filling process. YLJ-50 was used to press the mixed powder. During the pressing process, the pressing rate was about 20 MPa/min, and the pressure was held for 5 min at the highest pressing pressure. Afterward, it was slowly unloaded in order to reduce the residual stress in the specimen, and the produced blank was obtained by demolding. The preformed billet was placed on a crucible sprinkled with quartz sand and was then pushed into the middle of the tubular Nabertherm sintering furnace with a more uniform temperature. The sintering atmosphere was N_2_. During the sintering process, the temperature ranges of 25–328 °C and 328–375 °C were the uniform heating stage, and the heating durations were 3 h 40 min and 50 min, respectively. The temperature was maintained for 30 min at 375 °C. After the insulation, the temperature was lowered to 322 °C at a constant rate within 1 h, and the temperature was maintained for 30 min. Within 1 h, the temperature had cooled to 310 °C at a constant rate, and the temperature further cooled to 0 °C after 30 min. The static compression experiments and dynamic compression experiments at different temperatures and different strain rates were carried out on the reinforced Al/PTFE specimens prepared under different pressing pressures.

### 2.2. Quasi-Static Compression Experiment

The quasi-static compression experiment system consisted of a loading system, a measuring system, and a temperature control system. The loading system and measuring system were carried by the WDW 100GD universal testing machine. The temperature control system was undertaken within a high- and low-temperature test chamber, which was used for temperature control in the low-temperature experiment. The liquid nitrogen tank was used to cool the Al/PTFE specimen, and the minimum temperature could reach −150 °C in the low-temperature experiment. By adjusting the liquid nitrogen flow rate and the temperature control device of the high- and low-temperature test chamber, the quasi-static compression experiment could be carried out under the required temperature conditions.

The size of the reinforced Al/PTFE specimen used in this paper is 14 ± 0.1 mm in diameter and 5 ± 0.1 mm in length at room temperature (23 °C). The quasi-static compression tests (No.1–No.3), with a strain rate of 0.002 s^−1^, were carried out on reinforced Al/PTFE specimens with pressing pressures of 400 MPa, 600 MPa, and 800 MPa. The quasi-static compression tests of No.4–No.11 reinforced the Al/PTFE specimens with 200 MPa pressure were carried out at 23 °C, −20 °C, −30 °C, and −40 °C, respectively, with strain rates of 0.002 s^−1^ and 0.02 s^−1^.

### 2.3. Dynamic Compression Experiment

**Figure 1:**
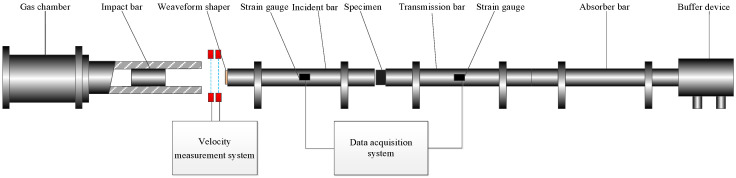
SHPB system.

The separation Hopkinson pressure bar (SHPB) device designed by Key Laboratory of Transient Physical Mechanics and Energy Conversion Materials of Liaoning Province was used for the dynamic loading experiment. As shown in Figure 1, the SHPB system consisted of a loading system, a velocity measurement system, a pressure bar system, a data acquisition system, and a low-temperature experimental system.

The impact velocity could be adjusted by changing the gas pressure in the gas chamber or the depth of the impact bar in the launch tube. Velocity was measured by laser and detonation velocity meter at the nozzle of the launch tube.

The pressure bar system included an incident bar, transmission bar, absorption bar, and buffer device at the end. Based on the resistance strain method, the electric signals generated by the strain gauges on the incident bar and the transmission bar were collected by the ultra–dynamic strain gauge, and the corresponding stress–strain data are obtained by software processing. The deformation process of the reinforced Al/PTFE specimen during dynamic loading was recorded by PCO.Dimax HS4 high–speed camera.

Polyurethane foam was used as a thermal insulation material for the low-temperature loading device, and a spirally wound copper tube was inserted into polyurethane foam in order to form a cooling chamber for low-temperature experiments. The temperature was controlled by adjusting the flow rate of liquid nitrogen in the copper tube.

The stress σ, strain ε, and strain rate ε’ of the specimen in the Hopkinson compression bar experiment are calculated based on the two–wave method. The calculation formula is as follows [33]:

(1) $$\sigma =\frac{AE}{A_{0}}\varepsilon_{t}$$

(2) $$\varepsilon =-\frac{2c_{0}}{l_{0}}\int_{0}^{t}\varepsilon_{r}dt$$

(3) $$\varepsilon^{\prime }=-\frac{2c_{0}}{l_{0}}\varepsilon_{r}$$

where *A* and *A*_0_ are the cross-section of bars and specimen, respectively; *E* is elastic modulus of bars; *c*_0_ is elastic wave velocity of bars; *l*_0_ is the length of specimen; *ε_r_* and *ε_t_* are the strains caused by reflected and transmitted waves, respectively.

Assuming the tested material was incompressible, the true stress $\sigma_{T}$ and true strain $\varepsilon_{T}$ of the material can be expressed as

(4) $$\sigma_{T}=\left(1-\varepsilon \right)\sigma$$

(5) $$\varepsilon_{T}=-\ln\left(1-\varepsilon \right)$$

### 2.4. Basic Parameters of Dynamic Compression Experiment

The reinforced Al/PTFE specimens with pressing pressures of 200 MPa, 400 MPa, 600 MPa, and 800 MPa were selected for dynamic compression experiments under three different strain rates, and the dynamic mechanical properties of the specimens under different loading pressures were analyzed. Table 1 shows the basic experimental parameters of the specimens prepared under different pressing pressures at room temperature.

## 3. Experimental Results and Analysis

### 3.1. Results of Quasi-Static Compression Experiment

**Figure 2:**
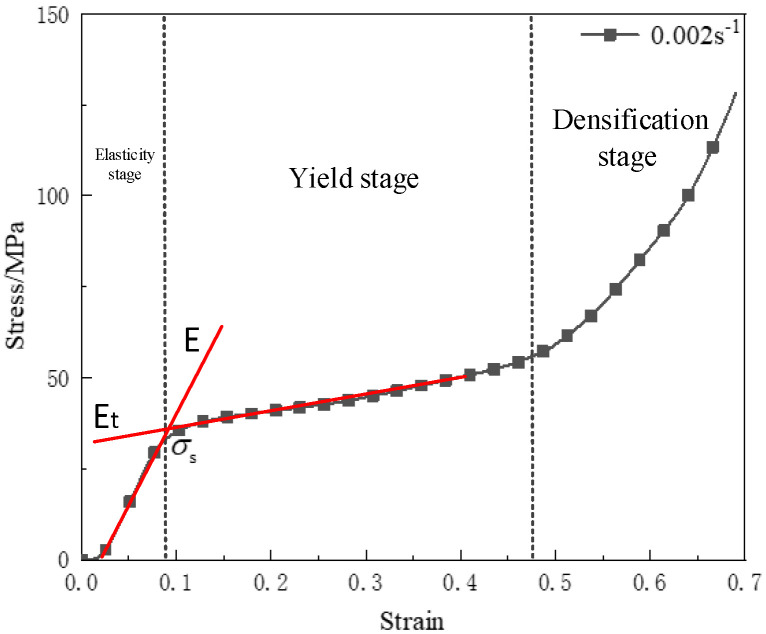
Schematic diagram of quasi–static compression performance parameters.

In order to characterize the quasi-static compressive properties of the reinforced Al/PTFE specimens, using the experiment of 200 MPa of pressing pressure at room temperature and a strain rate 0.002 s^−1^ as an example, the tangent slope of the stress–strain curve in the elastic stage was defined as the elastic modulus *E*, and the tangent slope of the stress–strain curve in the yield stage was defined as the hardening modulus Et. The stress value at the intersection of the two tangent lines was defined as the yield strength *σ_s_* [5]. The schematic diagram of quasi–static compression performance parameters is shown in Figure 2.

**Figure 3:**
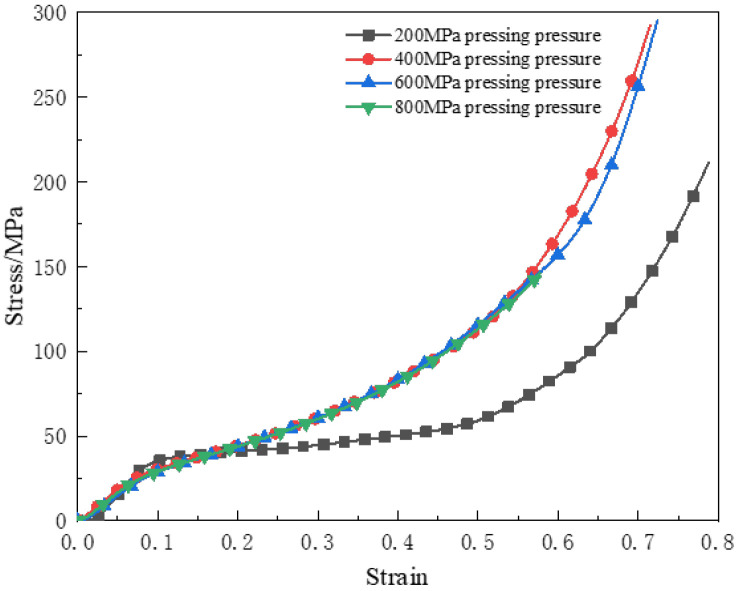
Quasi–static compressive stress–strain curves of different pressing pressures.

At room temperature, the stress–strain curves of the reinforced Al/PTFE specimens under different pressing pressures at 0.002 s^−1^ strain rate are shown in Figure 3.

It can be seen from Figure 3 that the pressing pressure had little effect on the elastic modulus and yield strength of the reinforced Al/PTFE specimen. The hardening modulus of the specimen prepared under 200 MPa pressing pressure was small, because there were more pores in the specimen under low pressing pressure. When the material entered the yield stage, the internal pores were crushed, and the specimen suddenly lost its bearing capacity. When the pressing pressure exceeded 400 MPa, the Al particles inside the specimen and the PTFE matrix were closely combined, and the stress–strain curves show a high degree of similarity.

**Figure 4:**
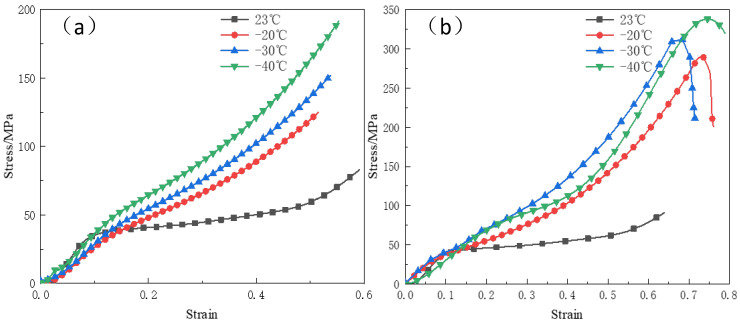
The stress–strain curves of quasi–static compression experiment under the same strain rate at different experimental temperatures (**a**) 0.002 s^−1^; (**b**) 0.02 s^−1^.

Figure 4 shows the quasi-static compression stress–strain curves of specimens under 200 MPa pressing pressure at different temperatures. The stress–strain curves of the reinforced Al/PTFE specimens show obvious temperature and strain rate correlation. As shown in Figure 4a, at the same strain rate, the elastic modulus, hardening modulus, and yield stress of the specimens all increased with the decrease of temperature. Under different temperature conditions, the material exhibited similar elastic behavior at the initial stage of deformation, and the elastic modulus and yield stress were not much different. However, when the strain exceeded 0.1, the hardening modulus increased significantly when the material entered the yield stage. The hardening modulus was 44 MPa at room temperature and 292 MPa when the temperature dropped to −40 °C. The decrease in temperature lead to the hardening behavior of the material, the decrease of toughness, and the decrease of the difference between the elastic modulus and the hardening modulus of the specimen. The viscoelastic property of the reinforced Al/PTFE material was weakened, and its deformation behavior is closer to that of the linear elastic material. By comparing Figure 4a with Figure 4b, when the temperature was the same, the elastic modulus, hardening modulus, and yield stress of the specimen all increased with the increase of strain rate, which has an obvious strain rate correlation.

It can be seen from the compressive stress–strain curves of the reinforced Al/PTFE specimens under different experimental conditions that the stress–strain curves can be roughly divided into the elasticity, yield, and densification stages. The material is a typical ductile material, and the yield point is not obvious, which is similar to the experimental results of Cai et al. [34].

### 3.2. Results of Dynamic Compression Experiment

#### 3.2.1. Effect of Pressing Pressure on Dynamic Compression Properties of Specimens

**Figure 5:**
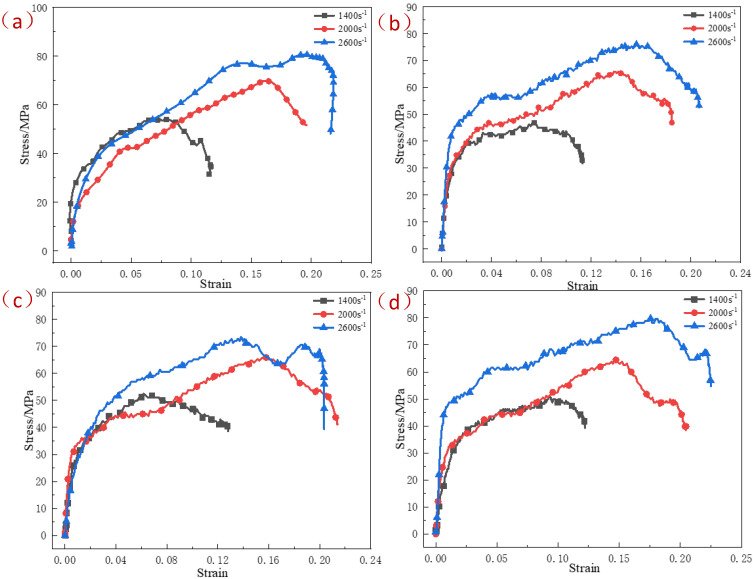
The stress–strain curves of specimens under the same pressing pressure and different strain rates at room temperature: (**a**) 200 MPa; (**b**) 400 MPa; (**c**) 600 MPa; (**d**) 800 MPa.

Figure 5 shows the stress–strain curves of specimens under the same pressing pressure and different strain rates at room temperature. It can be seen that under the same pressing pressure, the strain rate had a significant effect on the dynamic mechanical properties of the specimen, while the change of pressing pressure under the same strain rate had little effect on the dynamic mechanical properties of the specimen. With the increase of pressing pressure, the microstructure of the specimen became closer, but shear cracks were more likely to appear inside the specimen, and the residual stress was easier to release during the sintering process, which cannot guarantee the structural integrity of the specimen.

#### 3.2.2. Effect of Strain Rate on Dynamic Compression Properties of Specimens

**Figure 6:**
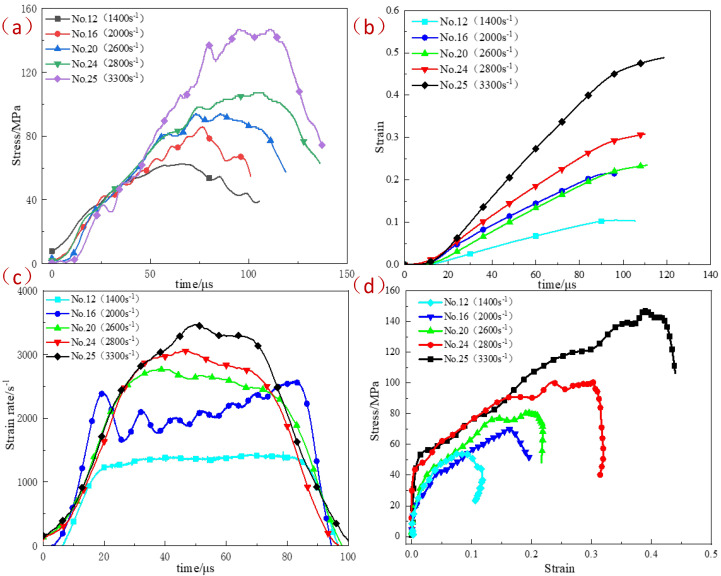
Experimental results of the reinforced Al/PTFE specimens under different strain rates at room temperature. (**a**) Stress–time curve; (**b**) strain–time curve; (**c**) strain rate–time curve; (**d**) stress–strain curve.

Figure 6 shows the stress–strain curves of reinforced Al/PTFE specimens under different strain rates at room temperature. At room temperature, the stress–strain curves at different strain rates show four stages: at the initial stage of loading, the stress increases rapidly with the increase of strain. The stress–strain curve at this stage is generally linear, and the elastic modulus is much larger than that of the specimen under quasi-static loading. This is due to the strain rate strengthening effect caused by the rapid increase of stress in the specimen, stress which has not reached the stress balance in the initial stage of stress wave loading. The load is primarily borne by the PTFE matrix in the initial stage. When the stress continues to increase and reaches yield stress at the matching strain rate, the specimen material yields, and the yield strength increases with the increase in strain rate. After the yield stage, the stress continues to increase with the increase of strain, and the material enters the strengthening stage. At this stage, the deformation is large, the PTFE matrix deforms sharply, and the Al particles squeeze and contact each other in order to form a force chain so that the specimen can bear higher stress. When the load exceeds the stress that the specimen can bear, the microcracks inside the specimen converge to form macro cracks, and the specimen fails.

At room temperature, the flow stress of the reinforced Al/PTFE material loaded with different strain rates increases with the increase of strain, a process which has an obvious strain–hardening effect. The dynamic yield strength of the materials increases with the increase of strain rate, and the material has an obvious strain rate-strengthening effect. The maximum stress of the stress–strain curve at each strain rate is defined as the peak stress, and the strain corresponding to the peak stress is defined as the peak strain. With the increase of the strain rate, the peak stress and peak strain of the dynamic compression of the specimen also increase monotonically. When the strain rate reaches 1400 s^−1^, the maximum true stress of the reinforced Al/PTFE material is 54 MPa, which is 1.5 times that of the traditional formula Al/PTFE [35].

**Figure 7:**
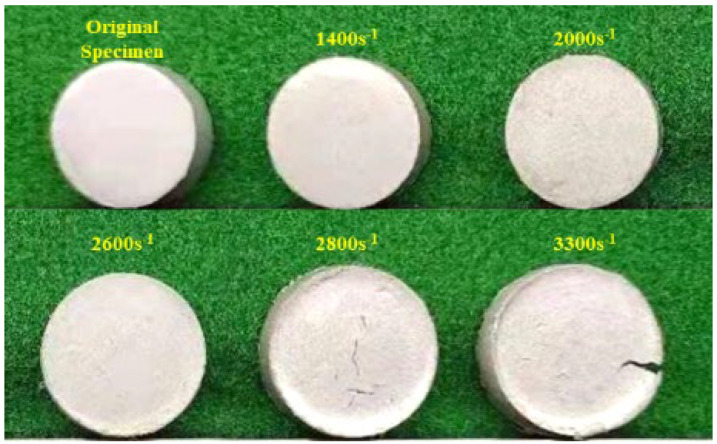
The specimens after dynamic compression at different strain rates at 20 °C.

Figure 7 shows the specimens after dynamic compression at different strain rates at 20 °C. With the increase of strain rate, the radial size of the specimen increased gradually. When the strain rate is low, the size of the specimen does not change much after the dynamic compression test, while the end face of the specimen increases uniformly with the multiple loading of the stress wave, and the end face is relatively flat. When the strain rate is high, the energy carried by the stress wave is large. Before the specimen reaches the stress equilibrium state, it exceeds the deformation response limit of Al/PTFE as a viscoelastic material, resulting in local stress concentration. As a result, the center of the specimen is rapidly compressed, forming a pit. When the strain rate is 2800 s^−1^, the PTFE matrix and Al particles debond, and cracks appear on the surface of the specimen under the action of tensile wave. When the strain rate reaches 3300 s^−1^, the cracks generated by the tensile wave of the specimen expand and converge to form an open crack.

#### 3.2.3. Effect of Temperature on Dynamic Compression Properties of Specimens

In order to determine the effect of temperature on the dynamic mechanical properties of the specimens, experiments No.26–No.31 were carried out. Table 2 shows the experimental parameters at different temperatures.

**Figure 8:**
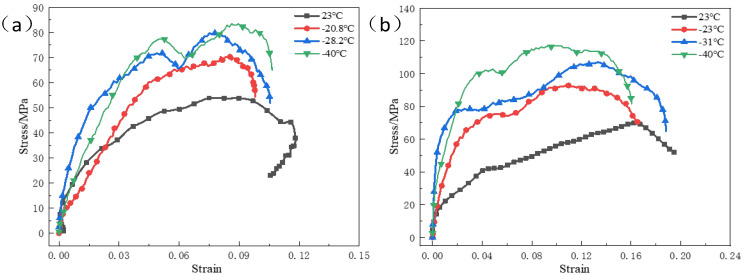
The stress–strain curves of reinforced Al/PTFE specimens at the same strain rate and different temperatures (**a**) 1400 s^−1^; (**b**) 2000 s^−1^.

Figure 8 shows the stress–strain curves of the reinforced Al/PTFE specimens at the same strain rate and at different temperatures. It can be seen from the figure that with the decrease of temperature, the peak stress of specimens under the same strain rate gradually increases, and the material presents characteristics of temperature softening, while there is no obvious corresponding relationship between peak strain and temperature variation. The effects of the same temperature and different strain rates on the peak stress and peak strain are compared. The results are shown in Table 3. At the same temperature, the peak stress and peak strain increase with the increase of strain rate. At low temperature, the stress has declines significantly after the yield stage of the material, which is a result of the stress unloading process.

### 3.3. Microstructure Characterization and Dynamic Compression Process of Reinforced Al/PTFE

**Figure 9:**
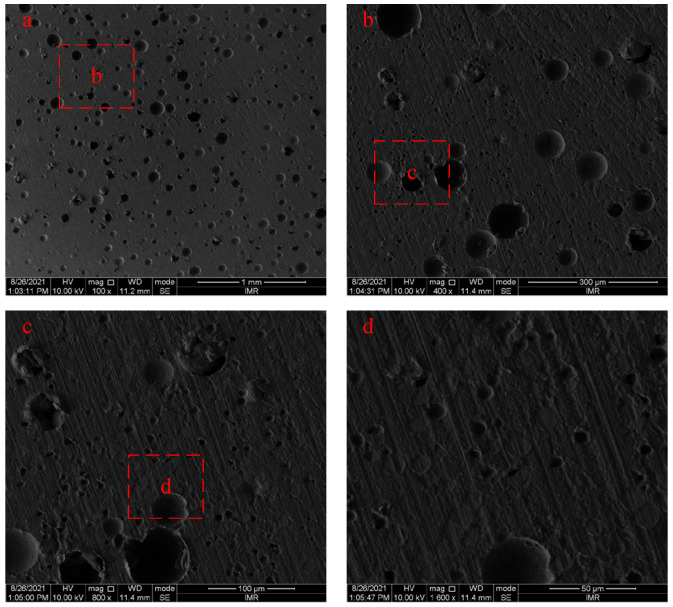
Microscopic morphology of the reinforced Al/PTFE specimen.

Scanning electron microscopy (SEM) was used to observe the microscopic morphology of the reinforced Al/PTFE specimens prepared in the same batch. The results are shown in Figure 9. The red box b in Figure 9 is the complete area of Figure 9, the red box c in Figure 9 is the complete area of Figure 9c, and the red box d in Figure 9 is the complete area of Figure 9. It can be seen from the figure that the Al particles were uniformly dispersed in the PTFE matrix without obvious agglomeration behavior. The reasons for some defects with apertures of 70–80 μm in the specimen are that PTFE has high crystallinity, and when the molding pressure is too large, the cold stretching generated by the powder particles may recover during the sintering process, resulting in the generation of micro-damages inside the material. The difference in thermal conductivity and thermal expansion coefficient between Al particles and the PTFE matrix will also lead to the existence of local large pores in specimens after sintering.

**Figure 10:**
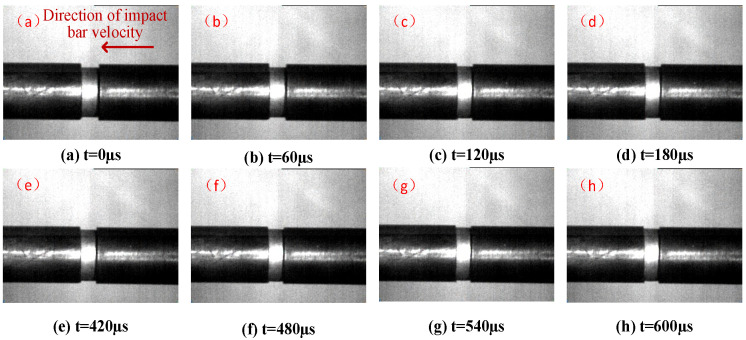
Experiment No.12 dynamic compression process.

The dynamic deformation process from experiment No.12 was captured by the high-speed camera with a frame rate of 167,711 fps. According to the two-wave method, the loading time of the first stress wave in No.12 was about 100 μs. In this paper, the compression deformation images of the specimen in the first two stress wave loading processes are selected, as shown in Figure 10. The previous frame’s image of the deformation of the specimen is defined as time t = 0 μs; (a)–(d) are the compression deformation process of the specimen loaded by the first stress wave pulse; (e)–(h) are the compression deformation process of the specimen loaded by the second stress wave. The results show that when the impact velocity is low, the overall deformation of the reinforced Al/PTFE specimen occurs under the action of stress wave. Since the friction force between the contact surface of the specimen and the bar cannot be completely eliminated, during the loading process, the radial stress existing on the end surface of the contact between the specimen and the bar hinders its radial deformation, which results in the specimen being drum-shaped during compression.

**Figure 11:**
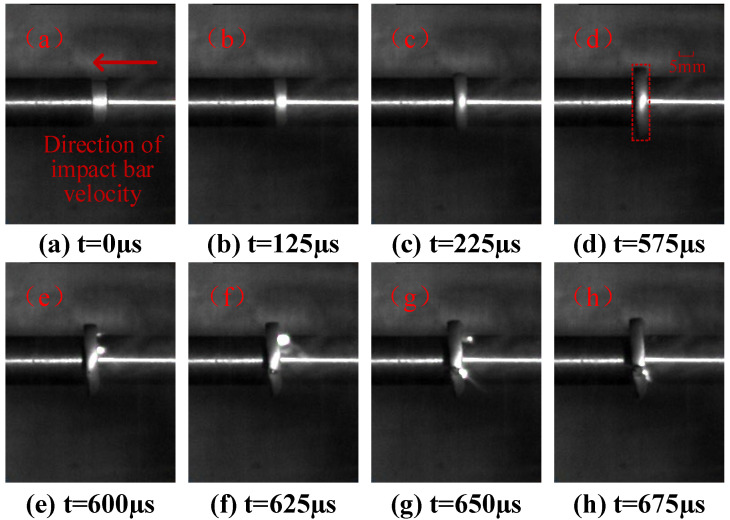
The impact ignition process of reinforced Al/PTFE.

Figure 11 shows the impact ignition process of the reinforced Al/PTFE specimen with the impact velocity of 35.2 m/s. Figure 11a–f shows the process of the specimen being gradually compressed to a pie shape. At t = 575 μs, although the diameter of the specimen has greatly exceeds that of the bar at this time, there are no cracks at the edge of the specimen. At this time, the diameter of the specimen is 23.7 mm, which is 1.69 times the initial diameter; the height of the specimen is 2.4 mm, which is 48% of the initial height. At t = 600 μs, the edge of the specimen cracks under the action of the tensile wave, but the overall structure is still relatively complete. At the same time, a shear band with a large strain gradient is formed in the contact area between the specimen and the edge of the compression bar. The material around the shear band undergoes an ignition reaction under the heat generated by plastic deformation and generates a bright spark [36].

## 4. Constitutive Equation of Reinforced Al/PTFE Active Materials at Room Temperature

From the stress–strain curves of the reinforced Al/PTFE material under quasi-static and dynamic compression, it can be seen that the material exhibits the characteristics of typical viscoelastic materials. In this paper, the mechanical behaviors of the reinforced Al/PTFE material are described based on the ZWT nonlinear viscoelastic constitutive model under different pressing pressures and different strain rates. The parameters of the constitutive equation of the material are fitted by the experimental results, and the accuracy of the model is verified.

### 4.1. ZWT Constitutive Model

**Figure 12:**
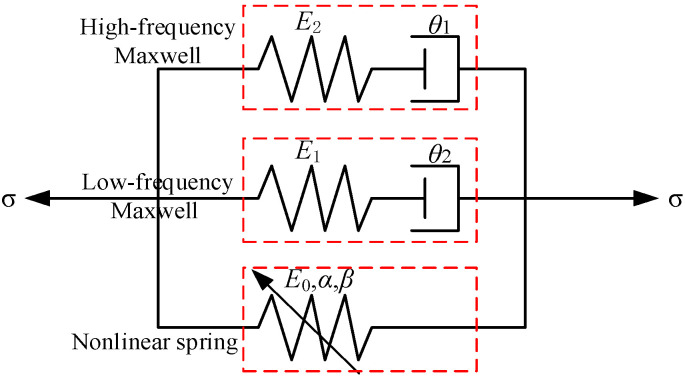
The rheological form of the ZWT model.

The ZWT viscoelastic model at strain rates of 10^−4^–10^3^ s^−1^ has been widely used in the study of polymer materials such as propellants, concretes, and organic glasses [37]. The rheological form of a ZWT nonlinear viscoelastic constitutive model consisting of a nonlinear spring and a low-frequency and high-frequency Maxwell viscoelastic element is shown in Figure 12.

The basic form of the ZWT constitutive model is

(6) $$\sigma =f_{e}\left(\varepsilon \right)+E_{1}\int_{0}^{t}\varepsilon^{\prime }\exp\left(-\frac{t-\tau }{\theta_{1}}\right)d\tau +E_{2}\int_{0}^{t}\varepsilon^{\prime }\exp\left(-\frac{t-\tau }{\theta_{2}}\right)d\tau$$

(7) $$f_{e}\left(\varepsilon \right)=E_{0}\varepsilon +\alpha \varepsilon^{2}+\beta \varepsilon^{3}$$

where $f_{e}\left(\varepsilon \right)$ describes the nonlinear elastic response of the equilibrium state independent of the strain rate, $E_{0}$ is the initial elastic modulus, and α and β are the corresponding elastic constants. The second and third integral terms in Formula (6) describe the low strain-rate and high strain-rate viscoelastic responses of two Maxwell elements, respectively. $E_{1}$ and $\theta_{1}$ are the elastic modulus and the stress relaxation time of the low-frequency Maxwell element, $E_{2}$ and $\theta_{2}$ are the elastic modulus and the stress relaxation time of the high-frequency Maxwell element. Since the stress–strain curve of the quasi-static compression experiment is linear before the yield stage, the equilibrium stress $f_{e}\left(\varepsilon \right)$ which is independent of the strain rate can be considered as $E_{0}\varepsilon$ only.

For quasi-static and low strain-rate loading conditions, the influence of the high strain-rate Maxwell element is ignored [38]. The quasi-static loading is a constant strain rate loading, $t=\varepsilon /\varepsilon^{\prime }$, and the equation is simplified to

(8) $$\sigma =E_{0}\varepsilon +E_{1}\theta_{1}\varepsilon^{\prime }\left[1-\exp(-\frac{\varepsilon }{\varepsilon^{\prime }\theta_{1}})\right]$$

Under high strain-rate loading conditions, the low-frequency Maxwell element and the two parallel springs describing the equilibrium stress element are reduced to a single spring element. SHPB experiment is constant strain-rate loading, $t=\varepsilon /\varepsilon^{\prime }$, and the equation is simplified to

(9) $$\sigma =\left(E_{0}+E_{1}\right)\varepsilon +E_{2}\theta_{2}\varepsilon^{\prime }\left[1-\exp(-\frac{\varepsilon }{\varepsilon^{\prime }\theta_{2}})\right]$$

### 4.2. ZWT Constitutive Model Considering Pressing Pressure

#### 4.2.1. Determination of Constitutive Model Parameters

**Figure 13:**
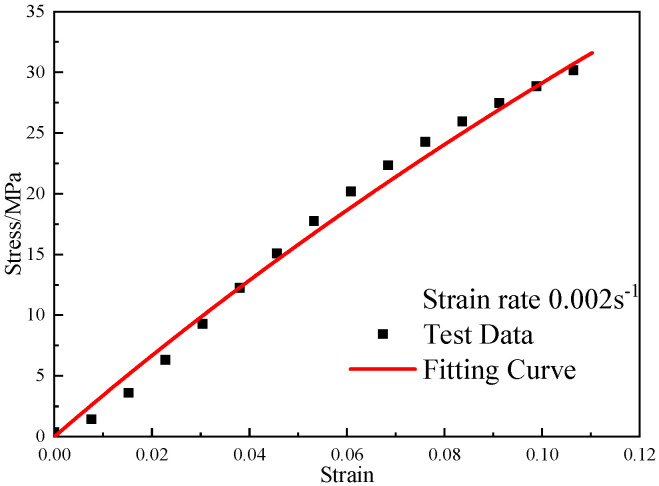
The fitting results of the ZWT constitutive model under low strain rate.

Due to the quasi-static loading, the static compressive stress–strain curve of reinforced Al/PTFE at the same strain rate was little affected by the pressing pressure factor. The stress–strain curve obtained from the quasi-static compression experiment with the pressing pressure of 400 MPa at room temperature was selected for fitting, and $E_{0}$, $E_{1}$ and $\theta_{1}$ were obtained. The fitting results are shown in Figure 13. The correlation coefficient $R^{2}$ of the curve fitting is 0.98. The fitting results are $E_{0}$ = 123 MPa, $E_{1}$ = 224 MPa, and $\theta_{1}$ = 83.7 s.

**Figure 14:**
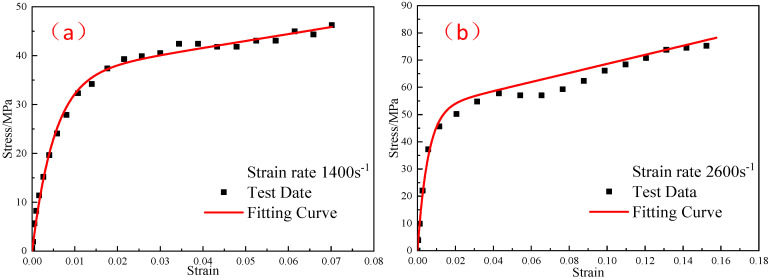
Comparison of the fitting results of the ZWT constitutive model with experimental results under 400 MPa pressing pressure and at different strain rates: (**a**) 1400 s^−1^; (**b**) 2600 s^−1^.

The stress–strain curves of No.13 and No.21 with different strain rates are fitted so as to obtain the elastic constant $E_{2}$ and relaxation time $\theta_{2}$, which is suitable for strain rates ranging from 1400 s^−1^ to 2600 s^−1^ when the pressing pressure is 400 MPa. The fitting results are shown in Figure 14. The correlation coefficients $R^{2}$ of curve fitting are 0.99 and 0.98, respectively. The fitting results are $E_{2}$= 719 MPa and $\theta_{2}$= 2.09 μs.

**Figure 15:**
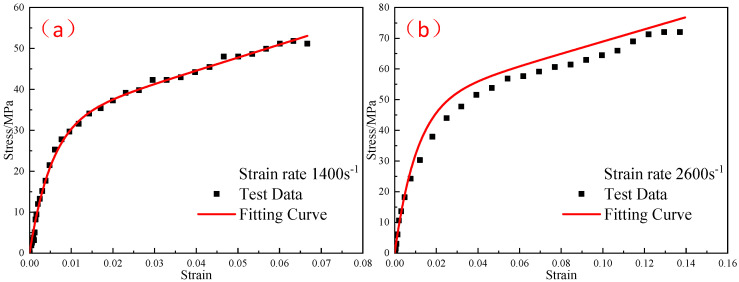
Comparison of the fitting results of the ZWT constitutive model with the experimental results under 600 MPa pressing pressure and at different strain rates: (**a**) 1400 s^−1^; (**b**) 2600 s^−1^.

The stress–strain curves of No.14 and No.22 with different strain rates are fitted so as to obtain the elastic constant $E_{2}$ and relaxation time $\theta_{2}$, which are suitable for strain rates ranging from 1400 s^−1^ to 2600 s^−1^ when the pressing pressure is 600 MPa. The fitting results are shown in Figure 15. The correlation coefficients $R^{2}$ of curve fitting are 0.99 and 0.94, respectively. The fitting results are $E_{2}$ = 4.9 GPa, $\theta_{2}$ = 4.06 μs.

**Figure 16:**
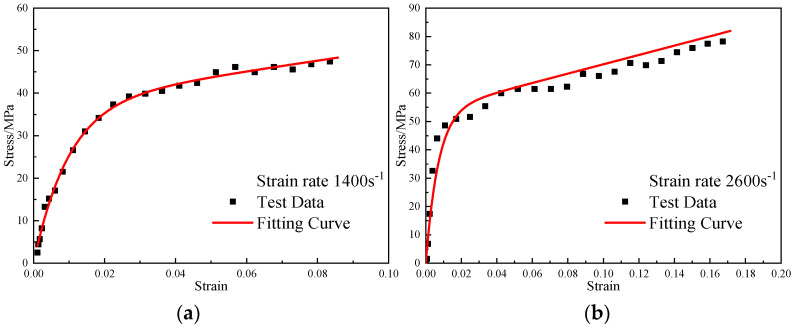
Comparison of the fitting results of the ZWT constitutive model with the experimental results under 800 MPa pressing pressure and different strain rates: (**a**) 1400 s^−1^; (**b**) 2600 s^−1^.

The stress–strain curves of No.15 and No.23 with different strain rates are fitted so as to obtain the elastic constant $E_{2}$ and relaxation time $\theta_{2}$, which are suitable for strain rates ranging from 1400 s^−1^ to 2600 s^−1^ when the pressing pressure is 800 MPa. The fitting results are shown in Figure 16. The correlation coefficients $R^{2}$ of curve fitting are 0.98 and 0.99, respectively. The fitting results are $E_{2}$ = 6.3 GPa and $\theta_{2}$ = 2.71 μs.

The parameters of the ZWT constitutive model obtained by different pressing pressures have been determined, and the parameters are listed in Table 4.

#### 4.2.2. Verification of Constitutive Model

**Figure 17:**
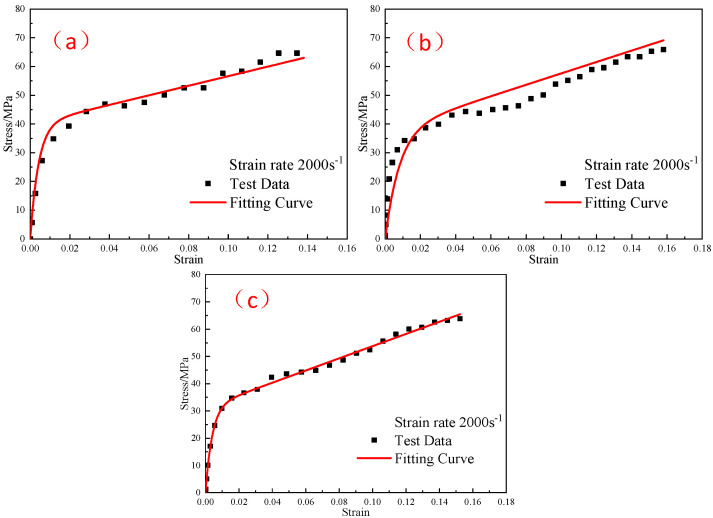
Validation of the reinforced Al/PTFE constitutive model considering the pressing pressure: (**a**) 400 MPa; (**b**) 600 MPa; (**c**) 800 MPa.

Experiments No.17–No.19, which were not involved in the model fitting, and whose strain rate is 2000 s^−1^, were used to test the ZWT constitutive model considering the pressing pressure in the range of 1400–2600 s^−1^. The results are shown in Figure 17. The correlation coefficients $R^{2}$ of curve fitting are 0.98, 0.94 and 0.99, respectively. In the corresponding strain rate range, the experimental curves are in good agreement with the predicted curves. The model can reliably describe the mechanical behavior of the reinforced Al/PTFE under the influence of pressing pressure factors in the strain rate range of 1400–2600 s^−1^.

## 5. Constitutive Equation of Reinforced Al/PTFE Active Materials at Low Temperature

The traditional ZWT constitutive model does not consider the influence of temperature on the material, which is slightly insufficient. Wang et al. [39] modified the ZWT constitutive model by adding temperature correlation terms, and described the mechanical properties of rubber at different temperatures and strain rates. Zhang et al. [40] improved the ZWT constitutive model based on temperature-dependent parameters and established a thermo-viscoelastic constitutive model to describe the mechanical properties of polyurethane films at different temperatures and strain rates. In order to describe the effect of the temperature on the mechanical properties of reinforced Al/PTFE materials, the constitutive model parameters were regarded as temperature-dependent functions.

### 5.1. ZWT Constitutive Model Considering Temperature Dependence of Parameters

#### 5.1.1. Establishment of the Constitutive Model

In order to describe the effect of temperature on the mechanical properties of reinforced Al/PTFE materials, the temperature parameter was introduced into the equation, and a simple thermal viscoelastic constitutive model was obtained.

(10) $$\sigma =E_{0}\left(T\right)\varepsilon +E_{1}\left(T\right)\theta_{1}\left(T\right)\varepsilon^{\prime }\left[1-\exp(-\frac{\varepsilon }{\varepsilon^{\prime }\theta_{1}\left(T\right)})\right]+E_{2}\left(T\right)\theta_{2}\left(T\right)\varepsilon^{\prime }\left[1-\exp(-\frac{\varepsilon }{\varepsilon^{\prime }\theta_{2}\left(T\right)})\right].$$

#### 5.1.2. Determination of Constitutive Model Parameters

**Figure 18:**
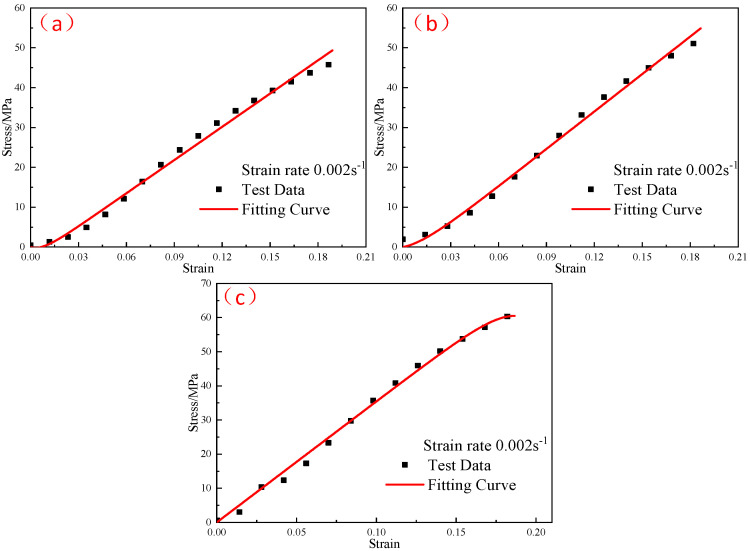
The results of the ZWT constitutive model fitting at low strain rates and different temperatures: (**a**) −20 °C; (**b**) −30 °C; (**c**) −40 °C.

In order to obtain the thermal viscoelastic constitutive parameters of the reinforced Al/PTFE materials, the stress–strain curves obtained by quasi-static compression experiments at three temperatures (−20 °C, −30 °C, and −40 °C) were fitted to obtain the elastic modulus $E_{0}$ and $E_{1}$ of the linear springs at three temperatures. The fitting results are shown in Figure 18, and the correlation coefficients $R^{2}$ of curve fitting are 0.97, 0.97, and 0.99, respectively.

**Figure 19:**
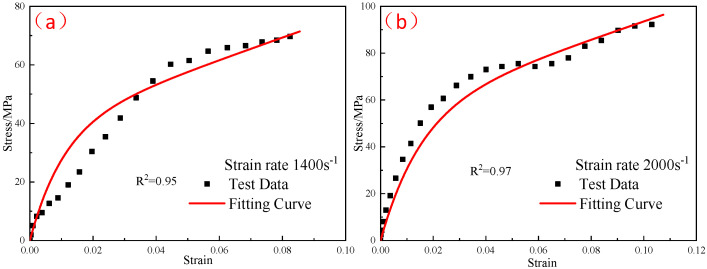
Comparison of the fitting results of the ZWT constitutive model with the experimental results under different strain rates at −20 °C: (**a**) 1400 s^−1^; (**b**) 2000 s^−1^.

**Figure 20:**
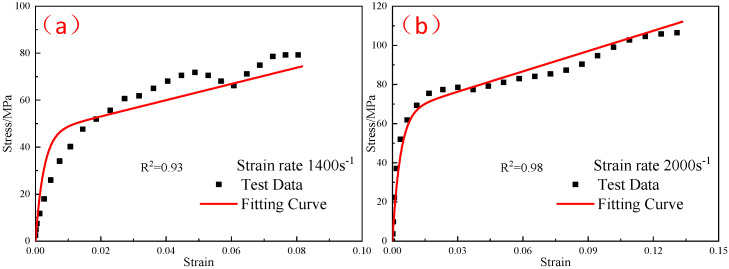
Comparison of the fitting results of the ZWT constitutive model with the experimental results under different strain rates at −30 °C: (**a**) 1400 s^−1^; (**b**) 2000 s^−1^.

**Figure 21:**
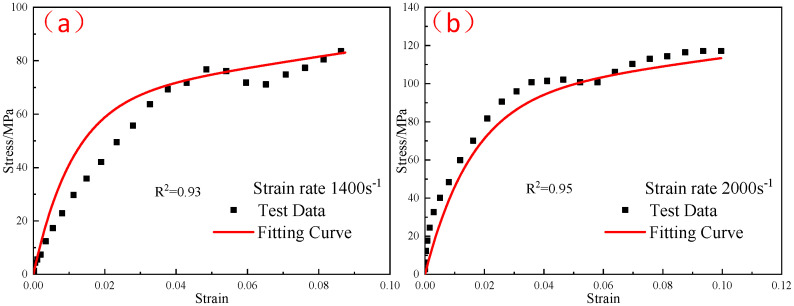
Comparison of the fitting results of the ZWT constitutive model with the experimental results under different strain rates at −40 °C: (**a**) 1400 s^−1^; (**b**) 2000 s^−1^.

The elastic moduli $E_{0}$ and $E_{1}$ of the linear spring obtained from the quasi-static compression experiment were substituted into Equation (10), and the elastic constant $E_{2}$ and relaxation time $\theta_{2}$ in the parameters of the ZWT constitutive model were obtained by fitting the experimental curves in each high strain-rate range at different temperatures. Figure 19, Figure 20 and Figure 21 shows the fitting results. The parameters of the constitutive model are listed in Table 5.

The parameters of the ZWT constitutive model in Table 5 are fitted concerning temperature factors in order to obtain the relationship between each parameter value and temperature, and the fitting results are shown in Table 6.

### 5.2. Consider the ZWT Constitutive Model with the Addition of Temperature-Dependent Terms

#### 5.2.1. Establishment of the Constitutive Model

Under the conditions of different loading strain rates, the yield strength of the reinforced Al/PTFE material decreases with the increase in temperature, which has a thermal softening effect. Referring to the basic form of the Johnson–Cook constitutive model [41], based on the above analysis, a simple thermal viscoelastic constitutive model was obtained by adding the thermal softening terms.

(11) $$\sigma =\left[E_{0}\varepsilon +E_{1}\theta_{1}\varepsilon^{\prime }\left[1-\exp(-\frac{\varepsilon }{\varepsilon^{\prime }\theta_{1}})\right]+E_{2}\theta_{2}\varepsilon^{\prime }\left[1-\exp(-\frac{\varepsilon }{\varepsilon^{\prime }\theta_{2}})\right]\right]\left[1-T^{*m}\right]$$

(12) $$T^{*}=\frac{T-T_{r}}{T_{m}-T_{r}}$$

where m is the thermal softening index, T is the current temperature, $T_{m}$ is the melting point of the material (set as the melting point of PTFE matrix 402 °C), and $T_{r}$ is the reference temperature (set as the lowest experimental temperature −40 °C).

#### 5.2.2. Determination of Constitutive Model Parameters

**Figure 22:**
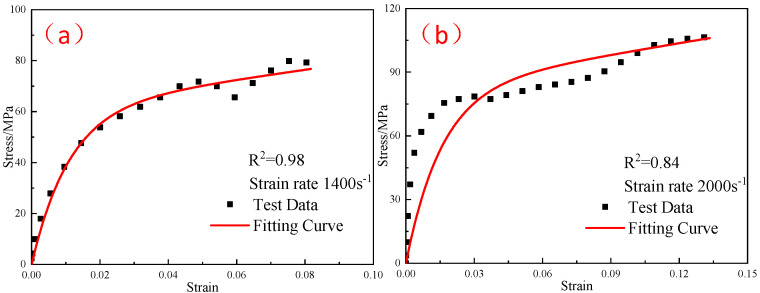
Comparison of the fitting results of the ZWT constitutive model with the experimental results under different strain rates at −30 °C: (**a**) 1400 s^−1^; (**b**) 2000 s^−1^.

**Figure 23:**
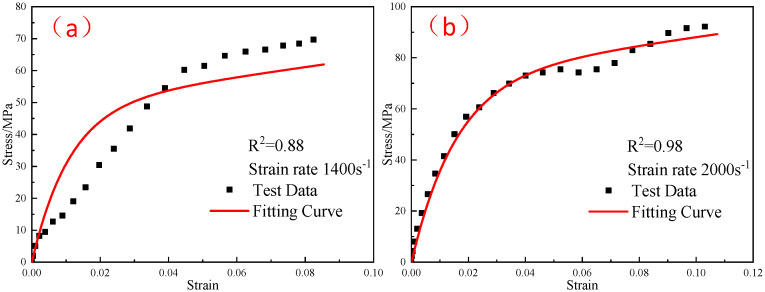
Comparison of the fitting results of the ZWT constitutive model with the experimental results under different strain rates at −20 °C: (**a**) 1400 s^−1^; (**b**) 2000 s^−1^.

**Figure 24:**
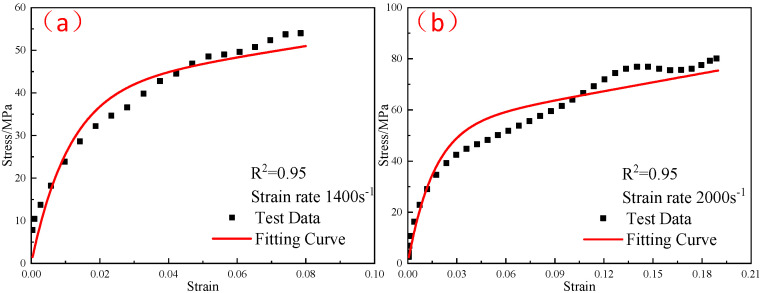
Comparison of the fitting results of the ZWT constitutive model with the experimental results under different strain rates at 23 °C: (**a**) 1400 s^−1^; (**b**) 2000 s^−1^.

The ZWT constitutive model parameters at −40 °C were substituted into Formula (11), and the thermal softening index m at different temperatures was obtained by fitting the dynamic stress–strain curves of the reinforced Al/PTFE at different temperatures. The fitting results are shown in Figure 22, Figure 23 and Figure 24. At −30 °C, −20 °C, and 23 °C, the thermal softening indexes m were 0.54, 0.47, and 0.48, respectively. The mean value of the thermal softening index m was set to 0.5.

## 6. Conclusions

Based on the traditional formula Al/PTFE (26.5%/73.5%), the reinforced Al/PTFE active material was prepared by cold pressing and sintering combining with rapid cooling. Quasi-static and dynamic compression experiments were conducted on the reinforced Al/PTFE specimens prepared under different compression pressures and at different temperatures and strain rates. Based on the compression experimental data, the parameters of the Zhu–Wang–Tang (ZWT) constitutive model of the reinforced Al/PTFE active material were fitted. The ZWT constitutive model parameters of the reinforced Al/PTFE active material under different compression pressures at room temperature and the ZWT constitutive model parameters of the reinforced Al/PTFE active material at low temperature were obtained. The accuracy of constitutive model parameters (elastic modulus, stress relaxation time, and thermal softening index) was verified. The ZWT constitutive model established in this paper considers both the temperature and the strain rate, making up for the shortcomings of the classical ZWT model (lack of a temperature term). The strength of this kind of active material has been greatly improved compared with the traditional process, which can be widely used in warhead shells, shaped charge liners, and other damage elements in order to meet the high efficiency damage demand for high-value targets. In the future, the application scope of temperature and strain rate will be broadened in order to improve the universality of the constitutive model. This paper provides theoretical and data support for the establishment of the ZWT constitutive model of active materials applicable to a wider range of temperature and strain rate.

## Figures and Tables

**Table 1:** The basic experimental parameters of the specimens under different pressing pressures at room temperature.

Experiment No.	Pressing Pressure (MPa)	Impact Speed (m/s)	Strain Rate (s^−1^)
No.12	200	8.2	1400
No.13	400	8.4	1400
No.14	600	9.0	1500
No.15	800	8.9	1500
No.16	200	13.9	2000
No.17	400	12.3	2200
No.18	600	13.8	2100
No.19	800	13.8	2200
No.20	200	18.1	2600
No.21	400	17.3	2500
No.22	600	16.2	2500
No.23	800	16.6	2700
No.24	200	24.3	2800
No.25	200	29.4	3300

**Table 2:** Experimental parameters under different temperature.

Experiment No.	Temperature (°C)	Impact Speed (m/s)	Strain Rate (s^−1^)
No.26	−20.8	8.4	1200
No.27	−28.2	8.4	1400
No.28	−40	8.1	1300
No.29	−23	13.4	2000
No.30	−31	15.1	2200
No.31	−40	13.3	2000

**Table 3:** Peak stress and peak strain corresponding to different strain rates at low temperature.

Temperature (°C)	Strain Rate (s^−1^)	Peak Stress (MPa)	Peak Strain
23	1400	54.0	0.08
23	2000	69.8	0.16
−20.8	1200	70.3	0.08
−23	2800	92.8	0.1
−28.2	1400	79.8	0.07
−31	2200	107.1	0.13
−40	1300	83.5	0.086
−40	2000	117.1	0.09

**Table 4:** Constitutive model parameters of the reinforced Al/PTFE prepared by different pressing pressures.

Pressing Pressure (MPa)	$E_{0}\;(\mathrm{MPa})$	$E_{1}\;(\mathrm{MPa})$	$\theta_{1}\;(s)$	$E_{2}\;(\mathrm{GPa})$	$\theta_{2}\;(\mu s)$
400	123	224	83.7	0.72	2.09
600	123	224	83.7	4.9	4.06
800	123	224	83.7	6.7	2.71

**Table 5:** Parameters of the reinforced Al/PTFE constitutive model at different temperatures.

Temperature (°C)	$E_{0}\;(\mathrm{MPa})$	$E_{1}\;(\mathrm{MPa})$	$\theta_{1}\;(s)$	$E_{2}\;(\mathrm{GPa})$	$\theta_{2}\;(\mu s)$
−40	319	242	0.7	5.9	12.7
−30	105	593	2.3	1.8	10.8
−20	53	631	3.4	1.6	7.61
23	238	49	1.7	4	3.68

**Table 6:** The relationship between the parameters of the reinforced Al/PTFE constitutive model and temperature.

Parameters	Fitting Equation	Correlation Coefficient
$E_{0}$	$20747-156T+0.29T^{2}$	0.96
$E_{0}$	$-36840+287T-0.55T^{2}$	0.91
$\theta_{1}$	$-194+1.48T-0.0027T^{2}$	0.99
$E_{2}$	$334-2.5T+0.0047T^{2}$	0.88
$\theta_{2}$	44−0.14T	0.93

## Data Availability

The data that support the findings of this study are available from the corresponding author upon reasonable request.
